# Automated amplicon design suitable for analysis of DNA variants by melting techniques

**DOI:** 10.1186/s13104-015-1624-8

**Published:** 2015-11-11

**Authors:** Per Olaf Ekstrøm, Sigve Nakken, Morten Johansen, Eivind Hovig

**Affiliations:** Department of Tumor Biology, Institute for Cancer Research, The Norwegian Radium Hospital, Montebello, Oslo, 0310 Norway; Institute of Cancer Genetics and Informatics, The Norwegian Radium Hospital, Oslo University Hosptal, Nydalen, Oslo, 0424 Norway; Department of Informatics, University of Oslo, Blindern, Oslo, 0318 Norway

**Keywords:** Amplicon design, Primer3, DNA variation, Mutation, DGGE, High resolution melting, Capillary electrophoresis

## Abstract

**Background:**

The technological development of DNA analysis has had tremendous development in recent years, and the present deep sequencing techniques present unprecedented opportunities for detailed and high-throughput DNA variant detection. Although DNA sequencing has had an exponential decrease in cost per base pair analyzed, focused and target-specific methods are however still much in use for analysis of DNA variants. With increasing capacity in the analytical procedures, an equal demand in automated amplicon and primer design has emerged.

**Results:**

We have constructed a web-based tool that is able to batch design DNA variant assay suitable for analysis by denaturing gel/capillary electrophoresis and high resolution melting. The tool is developed as a computational workflow that implements one of the most widely used primer design tools, followed by validation of primer specificity, as well as calculation and visualization of the melting properties of the resulting amplicon, with or without an artificial high melting domain attached. The tool will be useful for scientists applying DNA melting techniques in analysis of DNA variations. The tool is freely available at http://meltprimer.ous-research.no/.

**Conclusion:**

Herein, we demonstrate a novel tool with respect to covering the whole amplicon design workflow necessary for groups that use melting equilibrium techniques to separate DNA variants.

## Background

Friedrich Miescher discovered the nucleic acid in 1868–69 (reviewed by R. Dahm) [1, 2]. This may be defined as the starting point of a research field that has expanded into a huge area of medical and biological research. Many important methodological advances have been made in order to facilitate analysis of DNA. The polymerase chain reaction (PCR), first published by Kleppe et al. [3], which opened for mass amplification of DNA amplicons, is still a key method in modern molecular analysis of DNA. PCR amplification has the ability to amplify specific target sequences, as well as whole genomes up to a factor of 10^11^. In 1977, a DNA sequencing approach introduced by Sanger used dideoxy nucleotides to terminate enzymatic amplification of single stranded DNA [4]. This method was refined and eventually used to sequence almost the entire human genome. PCR amplification and Sanger sequencing, and variations of these core techniques, are still very important parts in present day DNA research. Following the development of these and other molecular methods, techniques enabling separation and visualization of amplified DNA have taken place, from the starting point of using radioactively labeled DNA and gel electrophoresis, through laser-induced fluorescence capillary electrophoresis, until various high throughput-sequencing platforms with the capacity to determine up to 5G bases/day.

One set of methods that was developed to detect unknown DNA variants was based on differential migration velocities of mutant single-stranded sequences or wild type/mutant heteroduplexes drawn through a macromolecular matrix by an electric field [5–7]. Of these, denaturing gel electrophoresis when performed in capillaries (constant denaturing capillary electrophoresis, CDCE) under optimized conditions has been demonstrated to comprehensively detect any point mutation, including single base insertions and deletions, in target sequences of ~70–140 bp [8, 9]. Methods employing CDCE have been reported with sensitivities to detect and identify mitochondrial and nuclear point mutations at levels at or above 2 × 10^−6^ mutations per gene copy in human cells, tissues and pooled blood samples [10–12]. Under a given concentration of chemical denaturant, such as urea, multiple capillary runs were required to define the generally narrow temperature range (~0.1 °C) that would separate heteroduplexes containing any of a wide variety of single deletion or single substitution mutants from wild type homoduplexes. CDCE has been adapted to commercial multi-capillary instruments, thus increasing the throughput of the method [13, 14]. A second improvement of the method was the introduction of oscillating temperature. By rapidly changing the denaturing condition in the capillaries, multiple DNA target sequences could be scanned simultaneously in the same run [13, 15–17]. One limitation of melting gel techniques results from DNA sequences rich on GC content, which can lose the resolution power due to complete strand dissociation at elevated temperatures. However, data from the complete melting map of the human genome indicates that the melting gel method can be applied to about 90 % of the human genome [18]. Because melting gel techniques are still much in use (more than 700 articles published in 2013), we have created a computational workflow that selects PCR primers [19], validates primer specificity in the genome of interest [20], and computes the DNA melting profile [21] with an artificial high temperature melting domain [22]. This web application, which is embedded in the Galaxy framework [23–25], will simplify amplicon design and increase the throughput of the method when amplicons are analyzed in multi-capillary instruments. Additionally, the amplicons designed in this web application, are also suitable for high resolution melting. This is a method that emerged after the introduction of thermocycler with fluorescent detection systems enabling visualization of the PCR amplification of DNA in real-time [26–30].

## Results and discussion

**Fig. 1:**
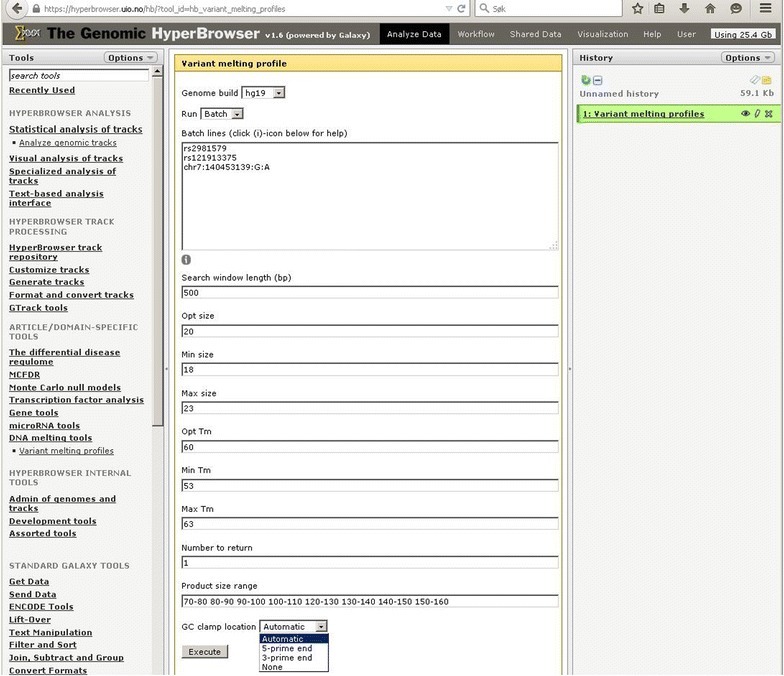
Screenshot of the web interface for Variant Melting Profile (http://meltprimer.ous-research.no/). Single or batch mode is selected in the “run” drop down menu. Design history can be saved in an optionally registered user account

**Fig. 2:**
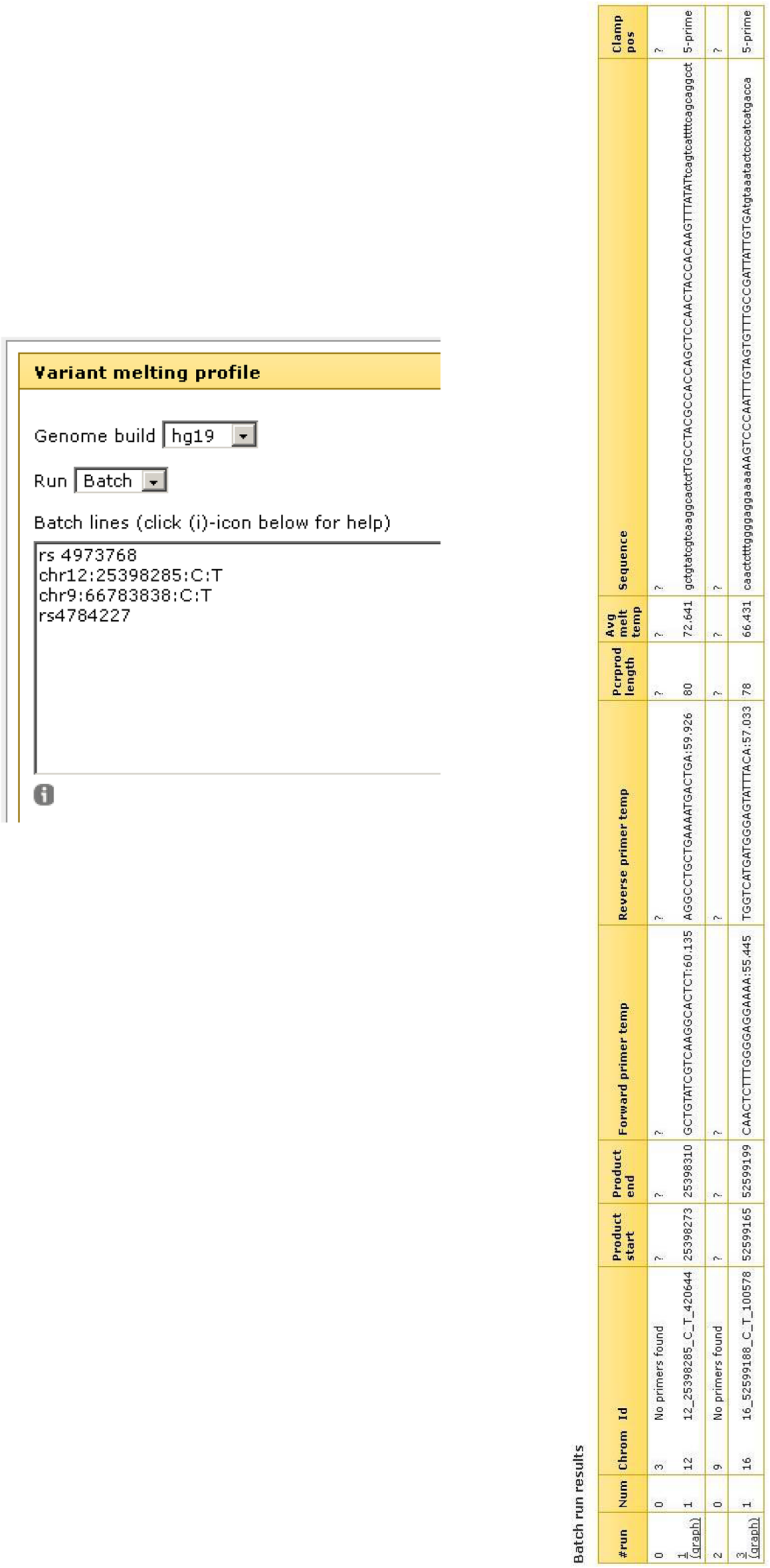
Output results when entering genomic variants in batch mode

The amplicon design interface is available at the following URL: http://meltprimer.ous-research.no/. Currently, the Variant Melting Profile tool supports design for DNA variants in the human genome (hg19) and the mouse genome (mm10). Upon request to the authors, additional genomes from other organisms can be added to the server. We have used the human genome as a model to test the Variant Melting Profile tool. The tool can run in *single* or *batch* mode, depending on the need to design amplicons for a single variant or a set of variants. There are two ways of entering variants of interest, either by using dbSNP reference IDs (e.g. rsIDs), or by specifying custom positions using a chromosome number, base pair position on the specific chromosome, and entering the relevant reference and variant alleles manually (chromosome:position:reference_allele:alternative_allele). An example of valid input is shown in Fig. 1. The lower variants entered in Fig. 1 demonstrate the two different ways of presenting the same genomic position. The resulting primers for these two ways of entering will be identical. The default values in the interface comprise standard primer length, annealing temperatures range and amplicon length intervals. These settings can be adjusted as needed. Depending on the allele discrimination method to be used downstream from the design, a high-temperature melting domain (GC-clamp) can be added to amplicons of interest. The GC-clamp is selected through a drop-down menu (Fig. 1). After the genomic position or reference numbers have been entered in the entry field, the workflow checks each position towards the latest build provided by NCBI. Un-recognized rs numbers will be called invalid and must be checked by the user, and invalid rs numbers must be removed prior to execution of the workflow. The execute button will start the selection of primers using Primer3, which is the most widely used open-source tool for selecting primers [19, 31]. From the primer list generated for each amplicon, the first candidate set is checked by an in silico PCR instance within the software for its specificity in the genome. Primer pairs resulting in more than one genomic region being amplified will be rejected. The column “ID” reports unspecific primers as “No primers found” (Fig. 2). The reason for this stringent setting is that amplicons with multiple hits in the in silico PCR are most likely to fail in the chemical PCR reaction. An example of a variant that will give multiple hits in the in silico PCR is chr9:66783838:C:T. The primers suggested, 5′AGACAGAAGCATTCTCAGGAACT3′ and 5′CCTGGTCTATCAAAAGAAAGGT3′, will result in 188 theoretical amplifications. Thus, the amplicon design tool rejected the primers and the amplicon.

**Fig. 3:**
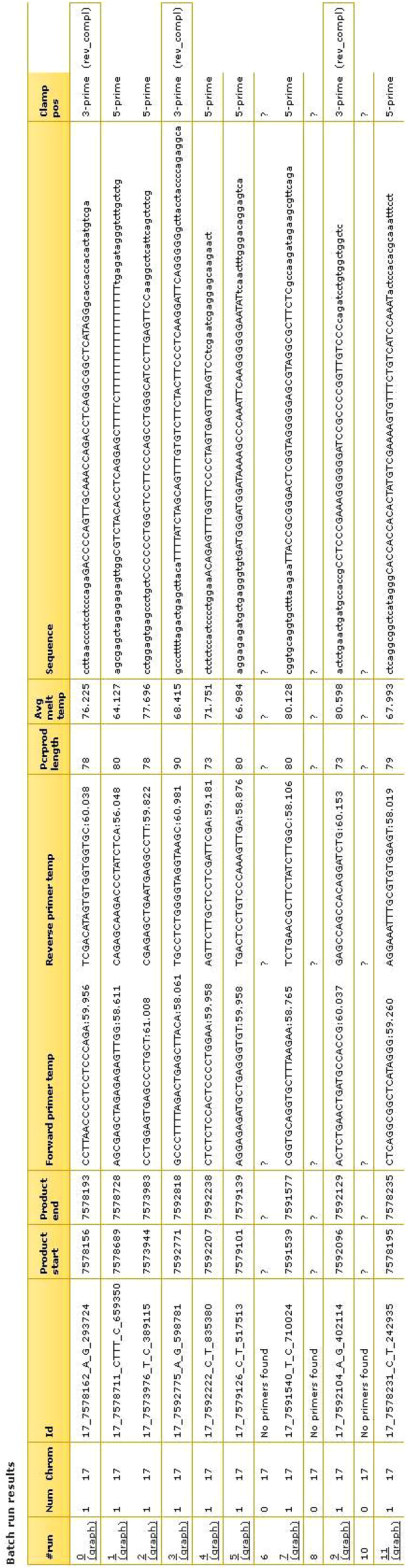
Part of results output when designing primers for 657 rs numbers in the TP53 gene

**Fig. 4:**
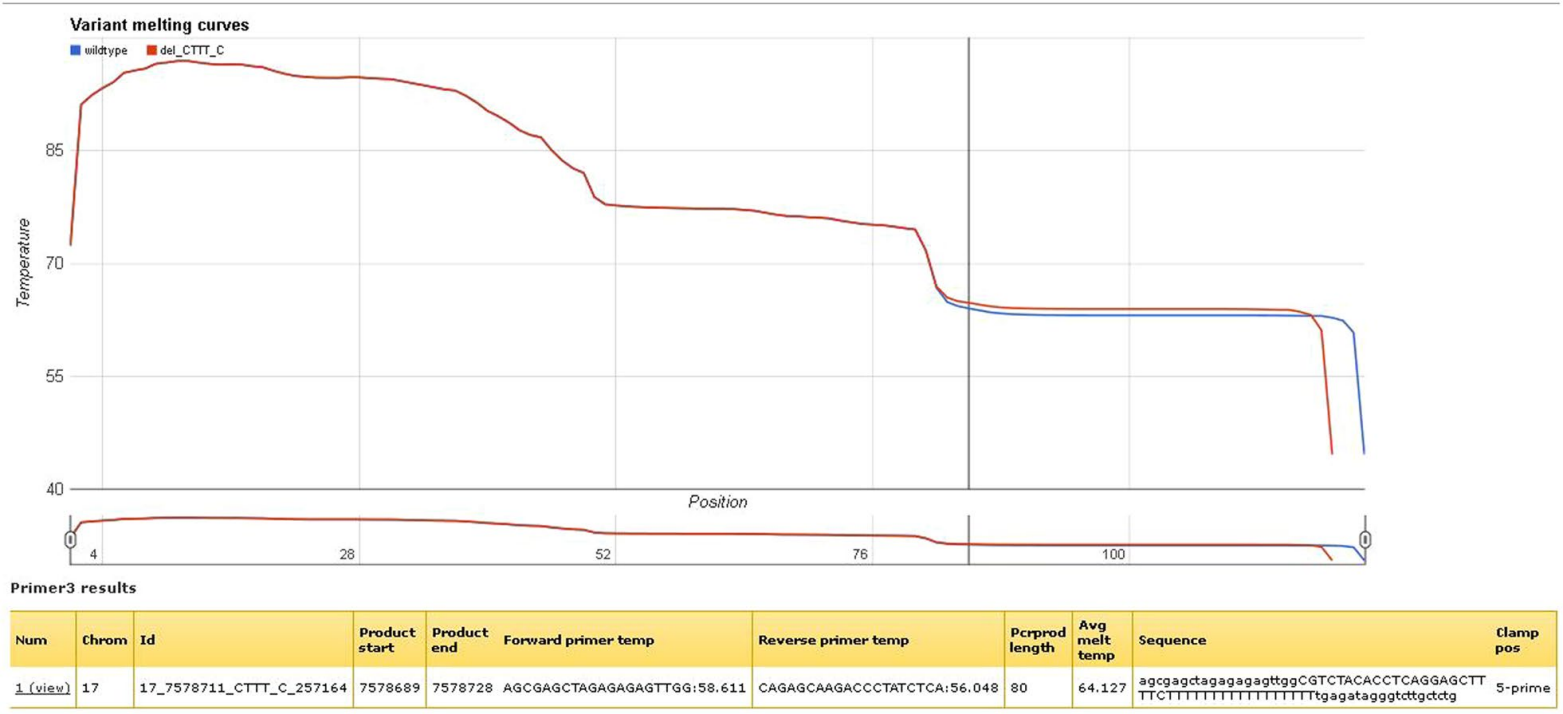
Screen capture of the result output. The melting profile of the amplicon is plotted as a function of base pair number in the amplicon. An artificial high temperature melting domain is extended onto one of the primers. The difference in melting between the two variants is shown. A zooming function allows for closer inspection of the melting profile. (https://hyperbrowser.uio.no/dev2/static/hyperbrowser/run_specific/036/36131/html/chart-0.html). The results table below summarizes the design, e.g. primers, fragment length, melting temperature and GC-clamp position

**Fig. 5:**
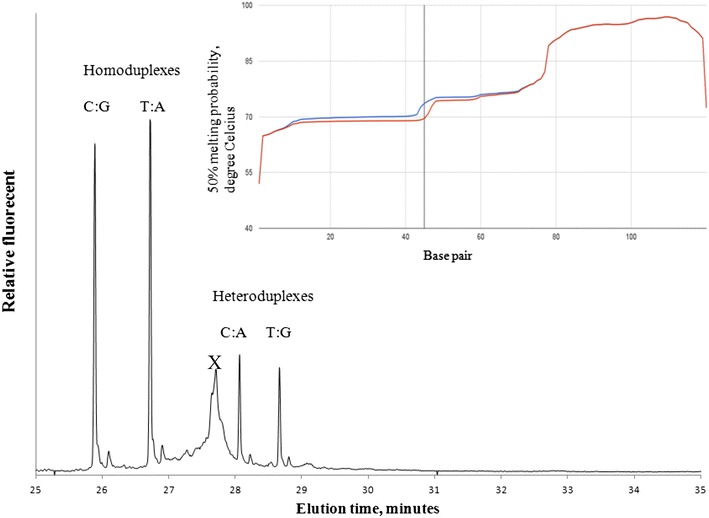
Allele separation of an amplicon with primers selected, controlled and simulated by the Variant Melting Profile. Inserted is the melting profile. Alleles were separated by cycling the temperature from 52–49 °C, 20 times during capillary electrophoresis. *Signal* was recorded by laser-induced fluorescence

**Fig. 6:**
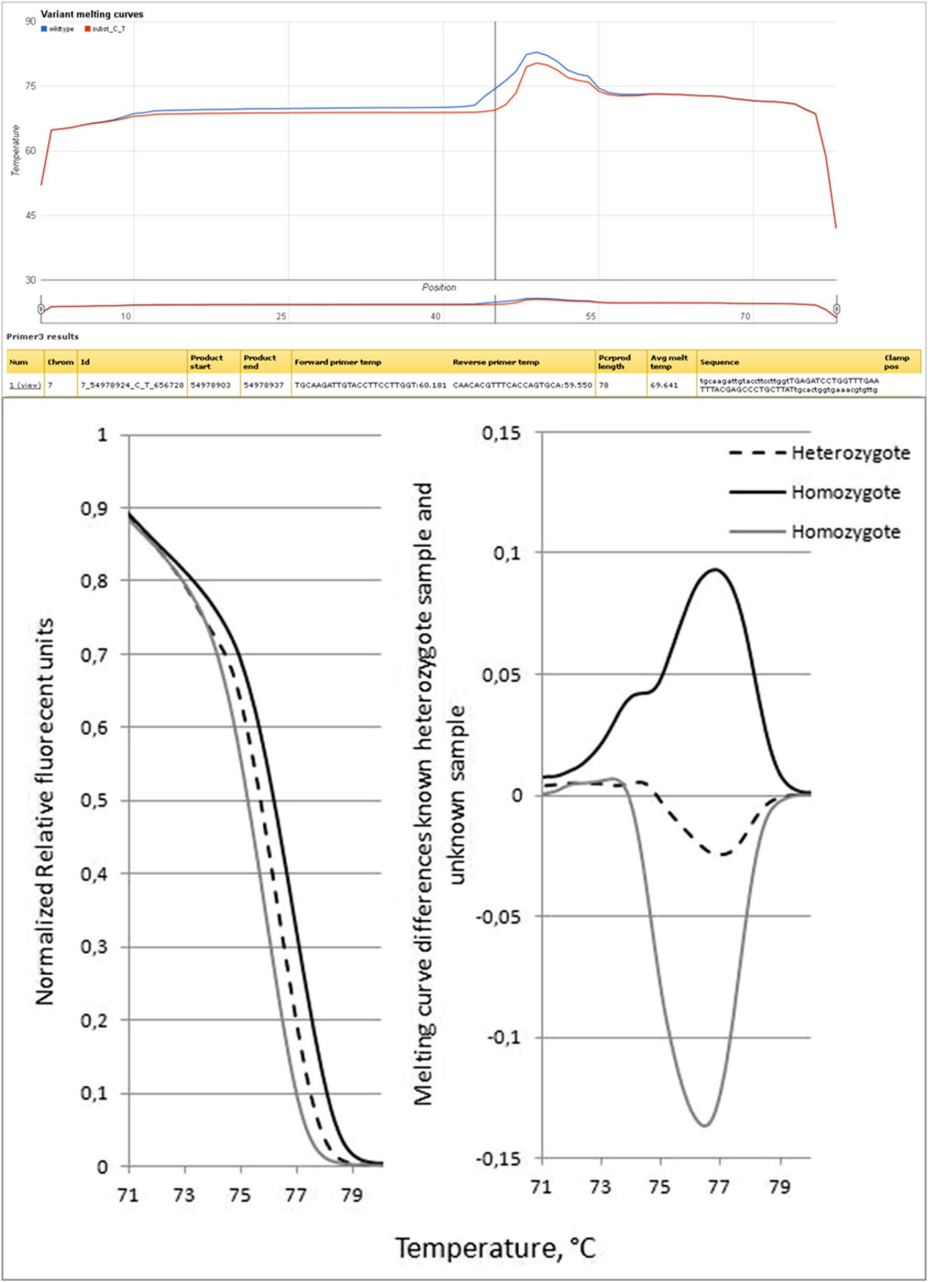
HRM analysis of different sample with different genotypes in the polymorphic site identified by rs2252586

 From the results list, useful information regarding amplicon length, sequence, mutation position and primer annealing temperature can be obtained. Figure 3 represents part of the results list when designing amplicons around variants in the TP53 gene (all data not shown). Of the 725 rs numbers in the input list, a total of 68 were found invalid and had to be removed before amplicon design could be performed. The computing time for primer selection, check of primer specificity, and melting curve calculation took 102 min for these amplicons combined. On average, one DNA melting assay is computed in 9.3 s. Importantly, manual design of primer sets is an error-prone process due to the large number of steps involved, especially in batch design settings. The default “orientation” of the GC-clamp is based on the amplicon melting profile. The clamp is simulated on the side of the amplicon with the highest average melting temperature, thus facilitating a decrease in melting temperature towards the side of the amplicon without the GC-clamp (Figs. 4, 5). Information on clamp position is found in the column “Clamp pos” (Figs. 2, 3). The melting temperature of amplicons with small insertions/deletions are always plotted from 5′–3′, with the GC-clamp positioned at the 5′ end. Consequently, the difference in melting profile of the deletion/insertion will be seen in the low melting domain and not in the GC-clamp (Fig. 4). The side is selected in such a manner that the melting temperature profile increases towards the GC-clamp. This is to avoid “dips” in the low temperature melting domain [16, 32], which would give peak broadening and loss of resolution. However, the tool also allows the user to determine the specific placement of the GC-clamp. As seen in Fig. 1, the drop-down menu for ‘GC clamp location’ has several options for placement of the GC-clamp: “none”, “automatic” (i.e. selecting the side with highest average melting temperature), “5-prime end” or “3-prime end”. This functionality will allow the user to explore the effect of the GC-clamp in relation to the melting behavior of the fragments. The result column “Avg Melt Temp” calculates the average melting temperature of the target sequence between the primers. When corrected for urea concentration in the polymer (−3 °C/1 M urea) [33, 34], this would yield the analysis temperature used in a capillary electrophoresis instrument. An example of the melting profile is shown in Fig. 4 and part of Fig. 5. The vertical line in the melting graph indicates the variant position (Figs. 4, 5, 6). The Google chart feature has sliders that allows the user to focus the graph at the X-axis (Fig. 5). A core feature of the Galaxy framework is that the results of each analysis are stored in a history. It is optional for the user to register an account that allows for storage of the amplicon design history (Fig. 1). An arbitrary variant (rs2252586) was used as a test on the amplicon design, subsequently followed by ordering of the suggested primers and analysis of the amplified amplicon by cycling temperature capillary electrophoresis and high resolution melting (HRM). The PCR reaction was optimized in a temperature gradient thermo cycler. The gradient used was from 52.2–60.2 °C. The optimal annealing temperature observed for this amplicon was approximately 4 °C lower than the theoretical value given in the results list, which is in accordance to general PCR protocols [35]. Figure 5 depicts the clear baseline separation of the alleles in the amplicon. The single strand (X) is recognized as a distribution, in contrast to the double stranded target with appropriate melting properties. As previously demonstrated, single stranded DNA will not significantly alter its position in the electropherogram with changes in the denaturing conditions [34]. To test the workflow further, primer sets for 2500 SNP reference numbers were also successfully designed in one run.

**Fig. 7:**
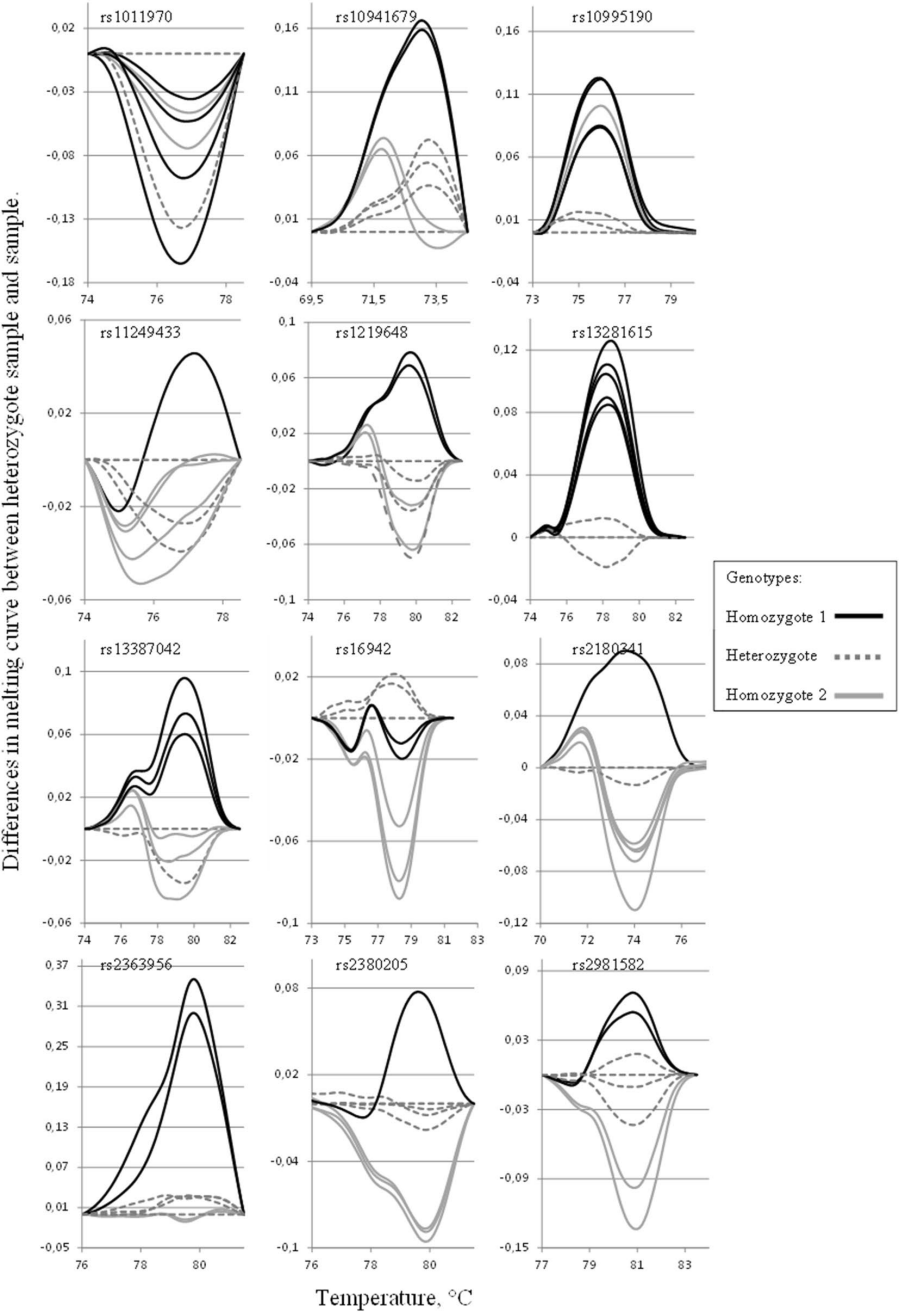
HRM assay of twelve polymorphic sites. The differences between a known heterozyote sample and seven DNA samples tested for unknown genotypes in twelve different amplicons is plotted for each amplicon. The amplicon id is given as rs number. Please note that the scale of the *x-* and *y-axis* is different for each plot. *Solid black line*, homozygote *1* sample, *dotted gray line*, heterozygote sample, *light gray line*, homozygote *2* sample

Figure 6 illustrates the melting profile of the amplicon (rs2252586) without the GC-clamp. The difference in melting temperature of the alleles is exploited in the HRM assay. By slowly heating the PCR product and recording the fluorescence for each temperature increment, a temperature dependent decrease in the signal occurs due to strand dissociation [26–30]. After normalization of the data and subtraction of the values of a known reference, genotypes can be visualized as in Fig. 6, lower part. To further explore the HRM assay, a set of twelve rs numbers (Table 1) previously analyzed by CTCE (manuscript in preparation) were subjected to analysis with Variant Melting Profile (batch mode) with default settings and no GC-clamp. For each amplicon, eight samples were subjected to real time-PCR following HRM. Figure 7 displays the analysis of the melting curve data, normalized and plotted as differences to a known heterozygote sample. The data suggest that HRM could be used in 8 or 9 amplicons as a genotyping tool. It is noteworthy that one amplicon (rs1011970) failed due to a second SNP (rs188512825) in the fragment. However, with better temperature control, HRM could in theory resolve the allele combinations of two neighboring SNPs (micro-haplotypes), given that the variants changes the DNA melting properties sufficiently.

**Table 1:** Reference SNP number and primers batch-designed and used in HRM assays

	Forward primer (5′-3′)	Reverse primer (5′-3′)
rs1011970	GGAAGATACAGGTGGAACTGGG	ACTGATAGGGAGCCAGCAGA
rs10941679	TATTTTAGACATGTTGACAT	TTTTATGCTGTGTTCTTTCC
rs10995190	CAATGGTTGTGTCCAAGTGCA	ACATACTGTTCTGATTGGCT
rs11249433	ATCAAATGAGTCACTGTGCT	AAAGCAGAGAAAGCAGGGCT
rs1219648	TCCCAAAACCAAAATTACTGA	AAGCCATGGCCATCCTTGAA
rs13281615	ACTCTTTTGATAAATTGGTAACT	CCCCAAACCCCCTACTCAGA
rs13387042	ACCAGAACAGAAAGAAGGCA	AGGAAGATTGAAGGAAGATTCGA
rs16942	CCTGAGCCAAATGTGTATGGG	GCTGTTTTTAGCAAAAGCGTCC
rs2180341	TCTGGACTCTCAATTCTATATCA	ACAAAGCTAAGGTAACAAGACA
rs2363956	ATGCAGAGGTGACAACAGGG	TTTCAAGGGGAACAGGGAGG
rs2380205	TGCCAATCTGTCCAGGAGGA	TTCCACCAGGCACGTTTCAG
rs2981582	ACTGCTGCGGGTTCCTAAAG	CCAGCACTCATCGCCACTTA

This is the first web application, to our knowledge, that combines the three features of primer selection around a DNA variant, controlling primer pair for specificity, and of computing the melting properties of the amplicon with or without a GC-clamp. Hence, this tool is novel with respect to covering the whole workflow necessary for groups that currently use melting equilibrium techniques to separate DNA variants.

## Methods

### Variant melting profile

The Variant Melting Profile is served as a tool within the Galaxy framework, which is an open source, web-based platform for data intensive biomedical research [23–25]. Galaxy is here used in combination with components from the Genomic Hyperbrowser [36, 37] as well as Google Charts for visualization (https://developers.google.com/chart/).

DNA variants (single nucleotide variants) can be given either as genomic coordinates (chromosome:position:reference_allele:alternative_allele), or as dbSNP reference IDs (e.g. rs9648696). User-specified parameters for selection of PCR primers (e.g. optimum primer sizes and primer melting temperatures etc.), as given by the Primer3 program [19] is used for finding suitable primers. The top candidate amplicon within the list of candidate primers is checked for specificity in the human genome using UCSC In-Silico PCR [38]. If the amplicon maps uniquely, a GC clamp can be added to the PCR amplicon, and the DNA melting profiles for the nucleotide variants are visualized in an interactive fashion. The melting profiles are generated using the thermodynamic model provided by Blossey and Carlon [39].

### DNA extraction, amplification and CTCE

Genomic DNA was extracted from anonymous blood donor samples by use of *GenoVision M48* extraction robot (Biorobot M48 station, Qiagen, Norway), following standard procedures as given by the instrument manufacture.

A 42 base pair sequence of dGTP and dCTP, also known as a GC-clamp, labeled with 6-FAM, was incorporated at one end of the amplified target using a set of three primers in the PCR setup. An amplicon was designed for analysis of DNA variation identified by NCBI SNP references number, rs2252586.

Labeled GC-clamp

5′6-fam-GCGCCCGCCGCGCCCCGCGCCCGTCCCGCCGCCCCCGCCCGGG3′

Reverse ½ GC-lamp tailed

5′CCCGCCGCCCCCGCCCGGG CAACACGTTTCACCAGTGCA3′

Forward

5′TGCAAGATTGTACCTTCCTTGGT3′

The PCR reaction mixture contained approximately 5 ng/µl genomic DNA, 0.4 mM dNTPs (0.1 mM of each dNTP) (VWR, Oslo, Norway), 1X Taq buffer, 0.075unit Taq/µl, 0.15 µM each of labeled GC clamp and the 1/2 GC-clamp tailed primer, while 0.3 µM of the “forward” primer (Integrated DNA Technologies, Leuven, Belgium) and 3 mM MgCl in a total reaction volume of 10 µl. Amplification was performed in a Eppendorf *Mastercycler ep gradient S* (Eppendorf, Hamburg, Germany) cycling 35 time between the temperatures of; cycles of denaturation for 10 s at 94 °C, annealing at 55.7 °C for 20 s and elongation at 72 °C for 30 s.

### Electrophoresis

Six-fam labeled PCR products were analyzed with a 96-capillary DNA analyzer, i.e. the MegaBACE™ 1000 DNA Analysis System (GE Healthcare Life Sciences, Pittsburgh, PA, USA). The instrument was modified to allow for elevated temperatures up to 65 °C. For detailed information about the modification, please contact the author P.O. Ekstrøm. Standard sequencing polyacrylamide (MegaBACE LPA) containing urea was replaced in the capillaries prior to each run. Samples were loaded into the capillaries from 96-well plates by electrokinetic injection at 161 V/cm for 15 s. The electrophoresis was carried out at a constant field of 145 V/cm. Laser-induced fluorescence was used with excitation at 488 nm (blue laser) and detection of emission at 520 nm (FAM channel). The scan rate was 1.75 Hz. The optimal separation temperature proposed by these programs was adjusted based on the urea concentration in the matrix. For each molar increment of urea, the temperature was lowered approximately 3 K (Kelvin) [40, 41].

The denaturing temperature in the capillary chamber, the cycling temperature, was programmed in the macro.ini file used by the Instrument Control Manager (ICM) software package (GE Healthcare Life Sciences, Pittsburgh, PA, USA). Data were converted to text files by MegaBACE Sequence Analyzer View and Edit software.

### Real time-PCR and high resolution melting (HRM)

A 25 µl Real time-PCR reaction was made up of 12.5 µl 2xMIX (PerfeCTa^®^ SYBR^®^ Green SuperMix, Quanta Biosciences, Gaithersburg, USA) 0.75 µl forward and 0.75 µl reveres primer with a concentration of 0.3 µM each. 2 µl DNA template and 9 µl H_2_O. The mixture was cycled 50 times with the following temperature, 95 °C for 15 s and 60 °C for 30 s. The fluorescence was read for each cycle, in a CFX Connect™ Real-Time PCR Detection System (Bio-Rad Laboratories AB, Oslo, Norway).

High resolution melting was performed after the temperature cycling by slowly increasing the temperature from 65–95 °C in increments of 0.1 °C. Fluorescent signal was recorded for 5 s after each 0.1 °C increment.

Primers used for the real-time PCR and HRM were selected by the Variant Melting Profile tool, checked for specificity by ePCR ordered from IDTDNA, with the following base composition: “Reverse” primer 5′CAACACGTTTCACCAGTGCA3′ and “forward” primer 5′TGCAAGATTGTACCTTCCTTGGT3′.

HRM curve data were normalized to the pre-melt (initial fluorescence) and post-melt (final fluorescence) signals. Thus, all samples were set to uniform, relative values from 100–0 %. The temperature axis of the normalized melting curves was shifted to the point where the entire double-stranded DNA was completely denatured. Differences in melting curve shape were further analyzed by subtracting the curves from a reference curve. We used a heterozygote sample as reference.

Following the initial test of HRM, 12 fragments were automatically designed on SNPs with references number given in Table 1. The fragments were subjected to Real time-PCR and HRM as described above, save for the annealing temperature which was set to 57 °C.

## Availability and requirements

Project name: MeltPrimer

Project home page: http://meltprimer.ous-research.no/

Operating system(s): Linux

Programming language: HyperBrowser/Galaxy framework, SQLite, Blat/gfServer/gfPcr, Primer3

License: GPL

Any restrictions to use by non-academics: no licenses needed.
